# The Role of Natural Polymorphic Variants of DNA Polymerase β in DNA Repair

**DOI:** 10.3390/ijms23042390

**Published:** 2022-02-21

**Authors:** Olga A. Kladova, Olga S. Fedorova, Nikita A. Kuznetsov

**Affiliations:** 1 Institute of Chemical Biology and Fundamental Medicine, Siberian Branch of Russian Academy of Sciences, 630090 Novosibirsk, Russia; fedorova@niboch.nsc.ru; 2 Department of Natural Sciences, Novosibirsk State University, 630090 Novosibirsk, Russia

**Keywords:** DNA repair, DNA polymerase beta, single-nucleotide polymorphism, protein–protein interaction, enzymatic activity, DNA repair coordination

## Abstract

DNA polymerase β (Polβ) is considered the main repair DNA polymerase involved in the base excision repair (BER) pathway, which plays an important part in the repair of damaged DNA bases usually resulting from alkylation or oxidation. In general, BER involves consecutive actions of DNA glycosylases, AP endonucleases, DNA polymerases, and DNA ligases. It is known that protein–protein interactions of Polβ with enzymes from the BER pathway increase the efficiency of damaged base repair in DNA. However natural single-nucleotide polymorphisms can lead to a substitution of functionally significant amino acid residues and therefore affect the catalytic activity of the enzyme and the accuracy of Polβ action. Up-to-date databases contain information about more than 8000 SNPs in the gene of Polβ. This review summarizes data on the in silico prediction of the effects of Polβ SNPs on DNA repair efficacy; available data on cancers associated with SNPs of Polβ; and experimentally tested variants of Polβ. Analysis of the literature indicates that amino acid substitutions could be important for the maintenance of the native structure of Polβ and contacts with DNA; others affect the catalytic activity of the enzyme or play a part in the precise and correct attachment of the required nucleotide triphosphate. Moreover, the amino acid substitutions in Polβ can disturb interactions with enzymes involved in BER, while the enzymatic activity of the polymorphic variant may not differ significantly from that of the wild-type enzyme. Therefore, investigation regarding the effect of Polβ natural variants occurring in the human population on enzymatic activity and protein–protein interactions is an urgent scientific task.

## 1. Introduction

Oxidation, alkylation, deamination, apurinization/apyrimidinization, and DNA strand break formation are only some of the many processes that lead to DNA damage [1,2,3,4,5]. Such DNA damage can initiate a malignant transformation of the cell. On the other hand, the same spectrum of damage occurs during chemotherapy and radiation therapy of cancers [6,7,8]. Therefore, the system of cell protection from damage—the enzymatic system of DNA repair—plays an important role in the processes of formation and treatment of cancer. 

**Figure 1:**
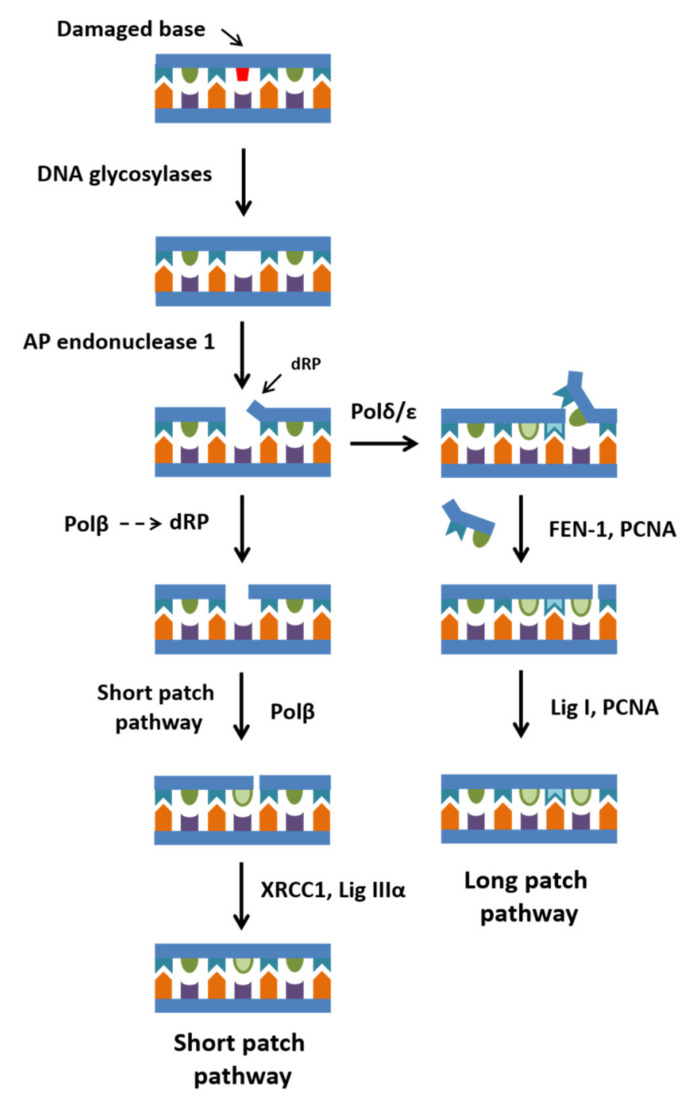
The scheme of short- and long-patch pathways of BER.

The initial stage in one of DNA repair pathways (base excision repair; BER) is implemented by DNA glycosylases that recognize various modified and mismatched bases and catalyze their removal (Figure 1). Then, apurinic/apyrimidinic endonuclease APE1 (AP endonuclease) incises the AP site remaining after the action of DNA glycosylases, resulting in the formation of 3′-hydroxyl and 5′-deoxyribose phosphate terminal groups. The main task of DNA glycosylases and AP endonucleases is to quickly and accurately find the location of a modified base or apurinic/apyrimidinic site among a huge number of intact bases and to initiate the repair process. 

Next, the intermediate DNA structure with a hydrolyzed AP site can be repaired by the short-patch or long-patch BER pathway [9,10]. In the short-patch pathway, DNA polymerase β (Polβ) adds only one nucleotide to the 3′ end of the hydrolyzed AP site, and then the dRP lyase activity of Polβ catalyzes β-elimination of the 5′-sugar phosphate residue, thus giving rise to a nick, which can then be repaired by ligase IIIα [11,12]. In the long-patch pathway, Polβ performs synthesis through strand displacement, generating a short flap DNA 2–10 nt long. This flap DNA structure is removed by endonuclease FEN1 [13,14]. After that, DNA ligase I repairs the break [9].

As BER is a multicomponent process, there is evidence of protein–protein interactions between participants of this repair pathway. Some interactions are aimed at increasing the rate of dissociation of enzymes tightly bound to an abasic site in duplex DNA [15]. Kinetic characterization of some of human DNA glycosylases has revealed that their product release is a rate-limiting step during the steady-state phase of the reaction [16,17,18]. Numerous studies have shown that APE1 promotes the dissociation of the DNA glycosylase–product complex, and this event, in turn, increases the multiple turnover rates of TDG, MBD4, ANPG, and OGG1 [19,20,21,22,23]. 

Multicomponent complexes with downstream proteins have been documented using cell extracts and recombinant enzymes in many studies. For example, Polβ also interacts with enzymes from BER, such as one of the bifunctional DNA glycosylases (NEIL1) [24], human AP endonuclease APE1 [25], XRCC1 [26,27,28], and PCNA [29] and PARP1 proteins. The XRCC1 has no enzymatic activity, and its main function in DNA repair is thought to promote the recruitment of other DNA repair proteins to the DNA damage site. The multiprotein complexes of XRCC1 with LigIIIα, Polβ, and PARP1 have been detected using different approaches [25,30,31]. 

The presence of Polβ, PNKP, and LigIIIα can enhance the interaction of XRCC1 with different DNA glycosylases [32,33,34,35]. The PARP1 protein can coordinate BER via direct interaction with some enzymes (APE1, PNKP, Polβ, LigIIIα, and TDP1) or indirect interaction mediated by the XRCC1 protein [36]. Using fluorescent titration methods, it has been shown that Polβ can form contacts with APE1 and PARP1 in the absence of DNA [36]. The interaction of PARP1, Polβ, and APE1 with nick-containing DNA was shown by photoaffinity labeling of BER proteins in a cell extract [37], indicating the interaction of these proteins during repair synthesis catalyzed by Polβ.

## 2. Functional Properties of Polβ

Polβ is considered the main repair DNA polymerase involved in the BER pathway, which plays an important part in the repair of damaged heterocyclic bases usually resulting from alkylation or oxidation [38,39,40]. In addition, Polβ participates in many other processes in the cell, namely, maintenance of the stability of the genome [41] and telomeres [42,43], meiosis [44], and nonhomologous end joining [45,46].

Polβ is a 39 kDa enzyme consisting of 335 amino acid residues encoded by the *POLB* gene located in the p11 region of chromosome 8 [47]. Polβ consists of one subunit, which can be divided into two domains by partial proteolytic cleavage. The 8 kDa N-terminal domain contains amino acid residues important for dRP lyase activity, and the 31 kDa C-terminal domain comprises amino acid residues necessary for nucleotidyl transferase activity.

For DNA synthesis, Polβ needs a template; for this purpose, the enzyme can bind to DNA of various structures. Although Polβ is capable of synthesizing DNA on a template consisting of a recessed DNA, nicked DNA, or gapped DNA, the enzyme is better at processing DNA containing small gaps with a 3′-hydroxyl group on the primer and a downstream 5′ phosphate that binds to the 8 kDa domain of the protein [48,49,50]. In a study on the Klenow fragment, reverse transcriptase and T7 RNA polymerase, a polymerase reaction mechanism, were proposed [51,52], additional evidence for which was provided by structural analyses of the Polβ triple complex with both substrates (DNA and dNTP) [53]. 

The active site of DNA polymerase contains a cluster of conserved amino acid residues carrying carboxyl groups and other polar amino acid residues at the polymerase active cavity (cleft). Carboxyl groups are critical for catalyzing the phosphoryl transfer reaction involving a nucleophilic attack by a 3′-hydroxyl group at the primer end on the α-phosphate of dNTP, releasing pyrophosphate.

**Figure 2:**
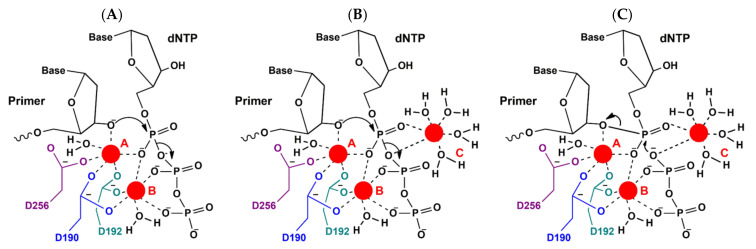
Proposed mechanisms underlying two- and three-divalent-metal-ion catalysis of nucleotidyl transfer (**A**,**B**) and facilitation of pyrophosphorolysis by a third divalent metal ion (**C**). The divalent metal ions are presented as red circles. (**A**) The two-divalent-metal-ion mechanism. The catalytic metal ion at site A is also coordinated by the 3′-OH of the primer, active-site carboxylate groups (Asp 190, 192, and 256), and a water molecule. The metal ion at site B is coordinated by active-site carboxylates (Asp 190 and 192), a water molecule, and nonbridging oxygen atoms of the β- and γ-phosphates. The 3′-OH of the primer is activated for a nucleophilic attack on the α-phosphate of the incoming dNTP. (**B**) The three-divalent-metal-ion mechanism. The C-site ion is coordinated by water molecules and the nonbridging oxygen atom of the α-phosphate and the bridging oxygen between α- and β-phosphates. The reaction proceeds as in (**A**) except that a third divalent metal ion at site C seems to stabilize the transition state or participates in the reverse reaction: pyrophosphorolysis. (**C**) Pyrophosphorolysis assisted by the third divalent metal ion. The third C-site divalent metal ion may help with the deprotonation and stabilization of O1 of PP_i_. This atom may then attack the nascent phosphodiester bond of the DNA backbone, and the primer 3′-hydroxyl can be protonated to restore the precatalytic active site for nucleotide incorporation.

In the proposed mechanism (Figure 2), these carboxyl groups coordinate two divalent metal ions, which then play a major role in catalysis [54]. One divalent metal ion (designated as Mg^2+^ ion B) promotes deprotonation of the 3′-hydroxyl group of the primer, while the other (Mg^2+^ ion A) stimulates the formation of a pentacovalent transition state of dNTP α-phosphate and departure of the leaving pyrophosphate group. The structure of the Polβ ternary complex [53] shows the coordination of two metal ions separated by ~4 Å (0.4 nm). 

Binding of the 5′-phosphate group is mediated by a lysine-rich 5′-phosphate–binding pocket located in the 8 kDa domain. This domain is essential for the activity of DNA polymerase because it increases DNA binding and polymerase processivity [55]. The general catalytic mechanism of Polβ follows the principle of sequential addition of substrates. First, Polβ binds to a DNA substrate and prefers DNA with short gaps containing a 3′-OH group and 5′-phosphate group. The enzyme then binds an incoming dNTP by preferably associating with the correct deoxynucleoside triphosphate, which is hydrogen-bonded to the template backbone. 

Upon the binding of dNTP, the enzyme undergoes conformational changes: considerable motion of subdomains as well as lower-amplitude conformational rearrangements of side chains [56]. Such conformational rearrangements cause the optimal arrangement of substrates for the direct nucleophilic attack of O3′ on αP of the incoming nucleotide, in accordance with the generally accepted catalytic mechanism of DNA polymerases, with two metal ions and three catalytic aspartate residues (positions 190, 192, and 256) [57]. After the chemical stage, at which nucleotidyl transfer occurs, a second conformational change associated with the opening of the subdomain becomes possible, where pyrophosphate is released and the product dissociates.

The proposed two-metal-ion mechanism has been considered universal for all DNA polymerases (Figure 2A). Nevertheless, for DNA polymerases from Y and X structural families, there is some evidence supporting the participation of a transient third divalent metal ion [58,59,60,61,62,63,64,65,66,67,68]. It is still not clear at which stage of the enzymatic process the third divalent metal ion appears—during nucleotidyl transfer [58,60,62,65] or product complexation [59,63,64]—or what its role is in the stabilization of the transition state (Figure 2B), in modulation of the chemical equilibrium of the nucleotidyl transfer through product state stabilization, in the catalysis of the reverse reaction (Figure 2C), or in product release.

Polβ is also active toward DNA molecules containing short gaps (2–6 nt), although its processivity is not as high as that of some other DNA polymerases and depends on the template DNA [48,69]. Polβ is capable of strand displacement synthesis, where it displaces the underlying DNA region [70]. This strand displacement synthesis is regulated in the cell via interactions with various proteins such as XRCC1, FEN-1, PARP1, APE1, and LigIII [25,71,72,73].

**Figure 3:**
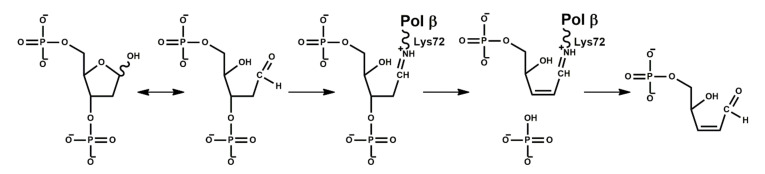
The mechanism of dRP lyase activity of Polβ.

In addition to its nucleotidyl transferase activity, Polβ possesses dRP lyase and AP lyase activities, and the former is more efficient (Figure 3) [12,74,75]. Deoxyribophosphate lyase activity (dRP lyase) is implemented by the 8 kDa domain. The Lys72 residue takes part in the formation of the Schiff base, and them the β-elimination reaction of the 3′-terminal phosphate group occurs. The 2,3′-unsaturated aldehyde is released from the 8 kDa domain.

## 3. Effects of Single-Nucleotide Polymorphisms (SNPs) on Polβ Activity

The expression of Polβ is essential for the cell’s response to the DNA damage that occurs during natural cellular processes. Defects in Polβ can lead to premature aging [76], cancers [77], and neurodegenerative diseases [78,79]. It is known that functionally deficient Polβ mutants have low efficiency of DNA repair, thereby, leading to a higher frequency of mutations in the genome.

Tumor cells carry significantly more mutations than somatic cells do, and the frequency of somatic mutations is not high enough to account for the number of mutations found in tumors [80]. To explain such a large number of mutations, it is assumed that cancer cells have a mutator phenotype [80]. This phenotype is believed to arise from mutations in genes encoding proteins that maintain genome stability [81]. 

One example in support of the mutator phenotype hypothesis is the discovery that mutation of certain genes in the DNA repair mismatch pathway results in hereditary nonpolyposis colon cancer [82]. It is reported that 30% of the human tumors that have been analyzed express proteins of polymorphic variants of Polβ, which are not found in normal tissue [77]. Some of these variants promote cell transformation and resistance to such chemotherapeutic agents as cisplatin [38,40,83]. 

The detected single-nucleotide mutations are not concentrated in any specific region of the protein and are localized in all subdomains of Polβ. It is known that mutations that affect the dRP lyase or polymerase activity of Polβ [84,85,86,87] reduce the efficiency of BER and cause hypersensitivity to alkylating or oxidizing agents. Some polymorphisms can lead to a substitution of functionally significant amino acid residues and therefore affect the catalytic activity of the enzyme and the accuracy of insertion of the desired nucleotide opposite the single-nucleotide gap formed in DNA. 

On the other hand, other known substitutions of amino acid residues are far away from the polymerase or lyase active sites of Polβ but are associated with various types of cancers. Such mutations may disrupt protein–protein interactions of Polβ with other proteins, for example, with enzymes involved in BER. It is known that Polβ does interact with enzymes from this pathway, for instance, with human AP endonuclease APE1 [25], XRCC1 proteins [26,27,28], and PCNA [29]. Nonetheless, it is still unclear what the effect of protein–protein interactions between BER participants is on the efficiency of damaged-heterocyclic-base repair in DNA.

SNPs represent the most common type of genetic variation in humans [88]. Genetic variation caused by SNPs, in particular nonsynonymous SNPs (nsSNPs) arising in protein-coding regions, alters the encoded amino acid and can induce structural and functional changes in the mutated protein. Not all of the structural and functional changes caused by an nsSNP are potentially destructive or harmful. Some nsSNPs influence structural properties, whereas others have functional implications. Databases contain information about more than 8000 SNPs in the gene of Polβ. 

Polymorphisms can lead to biochemical changes, BER deficiency, and predisposition to cancer [84,89,90,91,92]; therefore, an important and urgent task for researchers is to determine the impact of polymorphisms on cancer predisposition and to find possible reasons for this predisposition: a decrease in Polβ activity due to specific point mutations or the influence of these mutations on interactions with other proteins partaking in DNA repair. There are polymorphic variants of Polβ (containing substitutions of amino acid residues) that manifest a significant change in the enzymatic activity of this protein.

### 3.1. In Silico Prediction of Effects of Polβ SNPs

Currently, there are many bioinformatic approaches that allow predicting the influence of an SNP on protein function. Here, we analyzed known polymorphisms of Polβ to identify those that can have a damaging effect on Polβ function.

Information about known SNPs of Polβ was retrieved from the NCBI dbSNP database (http://www.ncbi.nlm.nih.gov/snp) (accessed on 20 February 2021). SNPs leading to an amino acid substitution (nonsynonymous or missense mutation) were tested in six software applications (SIFT (Sorting Intolerant From Tolerant) [93,94], PolyPhen (Polymorphism Phenotyping) [95], CADD (Combined Annotation-Dependent Depletion) [96], REVEL (Rare Exome Variant Ensemble Learner) [97], MetaLR [98], and Provean (Protein Variation Effect Analyzer) [99]) regarding the hypothetical ability to influence protein function.

Multiple resources were employed to predict the implications of SNPs to improve the reliability of the predictions and to obtain a list of SNPs that are predicted to have a negative impact in at least five of the six programs used. Finding a set of deleterious SNPs by only one predictive approach may not always be sufficient and useful because some SNPs that have an estimated effect close to a cutoff may turn out to be false predictions. 

After the checking in the programs, we selected those polymorphic forms of Polβ where amino acid substitution exerted a damaging effect (SIFT: deleterious/tolerated, PolyPhen: probably and possibly damaging/benign, CADD: likely deleterious/likely benign, REVEL: likely disease causing/likely benign, MetaLR: damaging/tolerated, and Provean: deleterious/neutral). Table 1 shows the chosen polymorphic variants in which the effect of an amino acid substitution was predicted to be damaging in at least five of the six programs. 

**Figure 4:**
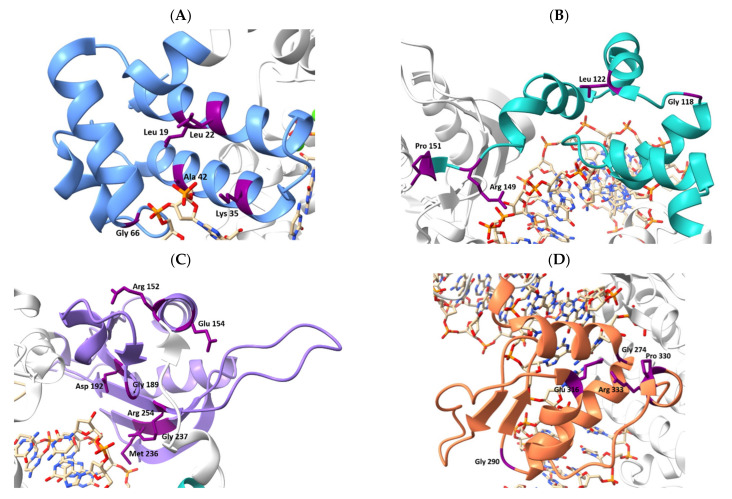
The locations of substitutions—caused by the SNPs predicted to be damaging—in Polβ structure (Protein Data Bank [PDB] ID: 7K96). (**A**) The 8 kDa dRP lyase domain is blue (**B**), the finger domain is green (**C**), the palm domain is light purple (**D**), and the thumb domain is orange. The affected amino acid residues are highlighted in dark purple.

Twenty-two such SNPs of Polβ were found (Figure 4). It should be noted that, among the listed predicted polymorphic variants, there are those for which supporting experimental data are available in the literature (for example, R152L, L22P, and K35E). As displayed in Table 1, the selected polymorphic variants represent substitutions of amino acid residues in various structural domains and functional regions of Polβ. Some mutations affect functionally important amino acid residues (e.g., Lys35 and Asp192), and some are located in the unstructured region of the protein (Pro330 and Arg333). A detailed analysis of known missense SNPs of Polβ is provided in Supplementary Table S1.

### 3.2. Cancers Associated with SNPs of Polβ

Reduced expression of Polβ in mice causes embryonic death [104], and embryonic fibroblasts obtained from such mice are insensitive to alkylating agents [14,105]. Downregulation of Polβ by small interfering RNA in a human cancer cell line enhances sensitivity to a chemotherapeutic agent [106,107]. On the other hand, Polβ overexpression in preclinical models is implicated in resistance to DNA-damaging agents [108,109]. 

It was recently demonstrated that a germline polymorphism of the *POLΒ* gene that encodes a Polβ variant with low catalytic activity induces cellular transformation and may be associated with increased susceptibility to cancer [110,111]. Approximately 30% of human tumors seem to express variant Polβ proteins that can induce cellular transformation in vitro. Moreover, mRNA expression of Polβ can also be impaired in some tumors, such as breast tumors [106]. 

For some SNPs, there is information in databases (COSMIC, HiveBiochemistry, and cBioportal) about the presence in various types of cancer (Supplementary Table S2) [78,83,84,85]. Mutations that affect the dRP lyase or polymerase activity of Polβ [84,85,86,87] are known to reduce the effectiveness of BER and cause hypersensitivity to alkylating or oxidizing agents. SNPs can lead to biochemical changes, BER deficiency, and predisposition to cancer [84,89,90,91,92]. 

Therefore, it is important to determine the effect of SNPs on predisposition to cancers and to identify possible causes of such predisposition: a decrease in Polβ activity due to a specific SNP or the influence of the SNP on interactions with other proteins involved in DNA repair. The predicted SNPs capable of strongly affecting Polβ function were checked by us for occurrence in various cancers according to databases cBioportal, HiveBiochemistry, and COSMIC (Table 2). The mutations causing amino acid substitutions at the same position as do the predicted SNPs, with a strong effect on Polβ function, were also added into Table 2.

The 10 amino acid substitutions of the 22 SNPs predicted to have a strong negative impact on Polβ function proved to have relevance to cancer. These data suggest that it is possible to predict the negative effect of some mutations that have not yet been detected in tumors.

### 3.3. Experimentally Tested Variants of Polβ

**Figure 5:**
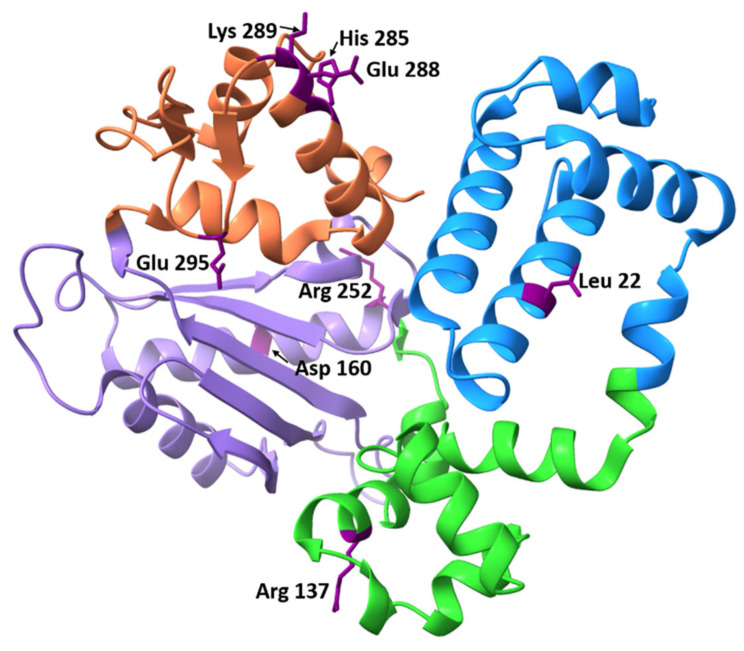
Locations of known experimentally tested Polβ SNPs in the protein’s structure (PDB ID: 7K96, DNA not shown). The dRP lyase domain is blue, the finger domain green, the palm domain light purple, and the thumb domain is orange. The substituted amino acids are highlighted in dark purple.

To date, experimental data on many polymorphic variants have been published. Most of these mutations have been found in tumors of patients with various cancers. The found SNPs affect different parts of the Polβ protein and alter Polβ function in different ways. The locations of known published SNPs are depicted in Figure 5.

#### 3.3.1. Glu295Lys

The polymorphic variant containing the Glu295Lys substitution does not possess polymerase activity, thereby, leading to the emergence of unfilled gaps in DNA, remaining for example after the action of AP endonuclease. This mutant of Polβ has been detected in patients with gastric carcinoma [84,112]. It was shown that mutant Glu295Lys binds to DNA containing a single-nucleotide gap as efficiently as does wild-type Polβ (the dissociation constants *K*_D_ are 28 and 12 nM, respectively) [84]. Mutant Glu295Lys also retains dRP lyase activity. 

Glu295 is located in the thumb subdomain of Polβ [113] . Based on X-ray-structural data, it can be assumed that, in the absence of DNA and deoxyribonucleotide triphosphates, the thumb subdomain is spatially closer to the palm subdomain, and residue Asp192 forms an ionic bond with Arg258, whereas Glu295 and Tyr296 engage in hydrogen-bonding interactions with the Arg258 residue. The Glu295Lys mutation significantly changes the polarity of the amino acid residue at this position, and it is possible that, due to this alteration, the interaction between residues Asp192 and Arg258 will be preserved. As a consequence, Asp192 cannot participate in the deoxyribonucleotide triphosphate transfer step.

#### 3.3.2. Leu22Pro

Another polymorphic Polβ variant associated with gastric cancer is Leu22Pro. It has been shown that this mutant does not possess the 5′-dRP lyase activity [102], which is necessary for the normal functioning of BER. Nevertheless, the Leu22Pro variant is able to fill gaps in DNA, that is, it has nucleotidyl transferase activity, as well as weakened affinity for DNA as compared to the wild-type enzyme. It is noteworthy that Leu22 is not a catalytic residue. This role is executed by Lys72 [114, but a substitution of this amino acid residue can eliminate the enzymatic activity. Residues important for DNA binding in the N-terminal 8 kDa domain are Lys41, Lys60, His34, Arg40, Tyr39, Lys 68, Lys 72, and Arg83 [114]. 

Although for Leu22, there are no structural details on the interaction with DNA, Leu is known to be an α-helix–stabilizing amino acid residue [115]. Thus, the Leu22 residue most likely contributes to the overall stability of the adjacent structural DNA-binding helix hairpin helix (HhH) domain (amino acid residues 55–79). Replacement of amino acid residue Leu22 (which stabilizes α-helix 1: residues 13–28) by Pro results in a several fold decrease in the efficiency of binding of the enzyme to DNA; therefore, if α-helix 1 is destabilized, then this alteration is likely to affect the DNA-binding site.

#### 3.3.3. Glu288Lys

There is a known polymorphic variant of Polβ, Glu288Lys, that is associated with rectal cancer and increases the frequency of mutations in A:T base pairs by threefold as compared to normal cells [116]. Polβ containing the Glu288Lys substitution does not differ in thermal stability and secondary structure from the wild-type enzyme. The dissociation constants *K*_D_ of the mutant and wild-type enzyme are also identical (19 ± 3 and 19 ± 1 nM, respectively) for the interaction with DNA containing a single-nucleotide gap. 

The polymerase reaction rate constant *k*_obs_ and the rate of product release *k*_ss_ are similar between these enzymes as well (*k*_obs_ = 8 ± 1 and 13.8 ± 0.5 s^−1^ and *k*_ss_ = 0.6 ± 0.2 and 0.68 ± 0.06 s^−1^ for wild-type Polβ and mutant Glu288Lys, respectively). The enzyme containing the Glu288Lys substitution has been shown to have lower fidelity on DNA containing a single-nucleotide gap opposite to adenosine. In addition, this loss of precision is specific to adenosine templates only because the results on DNA substrates containing other template bases are the same as those for wild-type Polβ [116].

#### 3.3.4. Arg152Cys

Another polymorphic variant found in patients with rectal adenocarcinoma is Polβ Arg152Cys. This substitution of arginine 152 with cysteine is of particular interest because of its location in the protein globule and its impact on the overall charge of the enzyme [55]. Additionally, Arg152 is known to be a methylation site in Polβ [117]. Analysis of tertiary structure of the polymorphic variant by circular dichroism spectroscopy has revealed that the structure does not differ from that of the wild-type enzyme. The activity of the mutant enzyme has also been tested. The polymorphic variant has a significantly lower polymerase activity when the primer is extended and the single-nucleotide gap in the DNA duplex is filled. 

Nevertheless, the dRP lyase activity and the ability to bind to DNA do not differ between the mutant Polβ and wild-type enzyme [38]. A possible reason is that the Arg152Cys mutation is located in the 31 kDa domain responsible for the polymerase activity of the enzyme, whereas the residues responsible for the dRP lyase activity and for DNA binding are situated in the 8 kDa domain. Experiments with nuclear extracts of HEK 293 cells expressing Polβ Arg152Cys and the wild-type enzyme indicate that the repair of DNA duplexes containing a uridine or tetrahydrofuran residue occurs less efficiently with Polβ Arg152Cys [38]. 

In that report, cells carrying the Polβ Arg152Cys variant accumulated more damage in genomic DNA when exposed to DNA-damaging agents. From these findings, it can be deduced that the Arg152Cys mutation reduces the biochemical activity of Polβ and may impair BER and contribute to genome instability and cancer. By contrast, when protein–protein interactions of Polβ Arg152Cys with other BER proteins were assessed, it was shown that the Arg152Cys substitution does not affect the interaction with enzymes APE1 and FEN1 and the protein PCNA.

#### 3.3.5. Arg137Gln

This is a known polymorphic variant of Polβ that has not yet been associated with any type of cancer. Nonetheless, this substitution is of interest because arginine at position 137 is located in the helix of the Polβ protein [113] and forms salt bridges with other adjacent amino acid residues. Replacement of the Arg by Gln leads to a loss of the positive charge and can result in substantial changes in biochemical and physiological properties of the enzyme. In addition, arginine 137 in Polβ is a site of methylation by the PRMT1 enzyme [39]. It has been found that the Arg137Gln mutation significantly reduces the polymerase activity (to 30% of the wild-type enzymatic activity), but at the same time, no changes are detectable in the course of dRP lyase activity and in the efficiency of binding to DNA because the Arg137Gln substitution affects the DNA polymerase catalytic domain (31 kDa domain) but not the dRP lyase (8 kDa) domain [118]. 

The Arg137Gln substitution has been reported to disrupt protein–protein interactions between Polβ and PCNA [118]. It has also been demonstrated that the Arg137Gln substitution impairs embryonic development in mice and increases sensitivity to DNA-damaging agents, such as H_2_O_2_ and methyl methanesulfonate [39].

#### 3.3.6. Asp160Gly

Some of the known polymorphic variants alter the polymerase activity of Polβ, for example, the Asp160Gly variant. This substitution is located in the palm subdomain and has been found in patients with renal carcinoma [99]. The substitution has been shown to increase the rate of the polymerase reaction of primer strand extension and filling of single-nucleotide gaps [119]. The greater efficiency of these reactions as compared to the wild-type enzyme may be attributed to the stronger DNA-binding affinity of the mutant enzyme [119]. To investigate the effect of this substitution on the sensitivity of cells to cisplatin, a Polβ variant called Asp160Gly was expressed in MCF-7 cells; it was demonstrated that the cells overexpressing Polβ Asp160Gly were more sensitive to cisplatin than are cells overexpressing wild-type Polβ.

#### 3.3.7. Lys289Met

This polymorphic variant correlates with rectal cancer. Residue Lys289 is located in the “fingers” subdomain, at the end of the α-helix (N α-helix). This α-helix is important for the closure of the finger domain when the correct nucleotide triphosphate binds. This variant more frequently attaches cytidine triphosphate opposite cytidine in DNA owing to poor discrimination of triphosphates during the transferase reaction. Lys289 forms an ionic bond (salt bridge) with the Gln324 residue in the ternary complex, and this interaction stabilizes the α-helix in the closed conformation. 

Substitution of lysine 289 with methionine can lead to a local alteration of interactions during the formation of the ternary closed complex [120]. It has been shown that the rate constant of nucleotide triphosphate incorporation into DNA containing a single-nucleotide gap in this variant of Polβ is significantly lower than that of the wild-type enzyme. At the same time, the dissociation constant *K*_D_ is virtually the same (1.3 ± 0.3 versus 2.3 ± 0.3 μM for Lys289Met and wild-type Polβ, respectively). Consequently, this substitution influences the stage of insertion of the correct nucleotide into DNA containing a gap owing to a decrease in the catalytic reaction rate constant, not because of the weaker affinity of the Polβ variant for DNA.

#### 3.3.8. His285Asp

This mutant of Polβ has been found in patients with rectal cancer [121]. A comparison of circular-dichroism spectra revealed that the general folding of the protein globule in the wild-type enzyme did not differ from that in the mutant [122]. It was reported that, under pre–steady-state conditions of the interaction of Polβ with DNA containing a gap, the observed reaction rate constants were similar between the wild-type and polymorphic variant (*k*_obs_ = 14 ± 2 versus 16 ± 1 s^−1^, respectively); a fast catalytic step of attachment of the nucleotide triphosphate to the DNA is followed by a slower step of the product release. 

The dissociation constants of the enzyme–DNA complex turned out to be comparable between the mutant and wild-type Polβ (9.2 ± 0.7 versus 6.7 ± 0.8 nM, respectively). Relative to the wild-type enzyme, the mutant possesses comparable fidelity (accuracy of incorporation of the correct nucleotide triphosphate into the DNA template). Histidine 285 is located in the α-helix (N α-helix) in the finger subdomain. The N α-helix spans amino acid residues 275–289, some of which are critical for Polβ fidelity. The N α-helix contacts DNA along the minor groove when Polβ is in a closed conformation (bound to both DNA and dNTP), and the formation of a pocket for dNTP binding becomes possible. 

His285 in the ternary complex is in close proximity to Lys289, and the nitrogen of the imidazole ring engages in a hydrogen bond with the carbonyl oxygen of the main chain of Ile323. The interactions of nitrogen atoms of Lys289 and His285 with the carbonyl oxygen of Ile323 appear to be important for keeping Polβ C-terminal unstructured amino acid residues 320–335 in place.

## 4. Conclusions

Our analysis of the literature indicated that SNPs in the *POLΒ* gene can have dramatic consequences. Some of the resultant amino acid substitutions are important for the maintenance of the native structure of Polβ and the contacts with DNA; others affect the catalytic activity of the enzyme or play a part in the precise and correct attachment of the required nucleotide triphosphate. 

On the other hand, the amino acid substitutions in Polβ can affect interactions with other proteins, for example, with enzymes involved in BER, while the enzymatic activity of the polymorphic variant may not differ significantly from that of the wild-type enzyme. Moreover, both these and other SNPs can correlate with various types of tumors in patients. Therefore, investigation regarding the effect of Polβ mutations occurring in the human population regarding enzymatic activity and protein–protein interactions is an urgent scientific task.

## Figures and Tables

**Table 1:** The list of Polβ SNPs capable of strongly altering the function of the DNA polymerase as predicted by at least five programs (SIFT, PolyPhen, CADD, REVEL, MetaLR, and/or Provean).

	SNP	SIFT	PolyPhen	CADD	REVEL	MetaLR	Provean	Refs. for Experimental Confirmation
1	L19P	Deleterious	Probably damaging	Likely deleterious	Likely disease causing	Damaging	Deleterious	
2	L22P	Deleterious	Probably damaging	Likely deleterious	Likely disease causing	Tolerated	Deleterious	[100,101,102]
3	K35E	Deleterious	Probably damaging	Likely deleterious	Likely disease causing	Tolerated	Deleterious	[75]
4	A42T	Deleterious	Probably damaging	Likely benign	Likely disease causing	Damaging	Deleterious	
5	G66R	Deleterious	Probably damaging	Likely benign	Likely disease causing	Damaging	Deleterious	
6	G118V	Deleterious	Probably damaging	Likely deleterious	Likely disease causing	Damaging	Deleterious	
7	L122R	Deleterious	Probably damaging	Likely benign	Likely disease causing	Damaging	Deleterious	
8	R149I	Deleterious	Possibly damaging	Likely deleterious	Likely disease causing	Tolerated	Deleterious	
9	P151L	Deleterious	Possibly damaging	Likely benign	Likely disease causing	Damaging	Deleterious	
10	R152L	Deleterious	Probably damaging	Likely deleterious	Likely benign	Damaging	Deleterious	[103]
11	E154A	Deleterious	Probably damaging	Likely deleterious	Likely disease causing	Damaging	Deleterious	
12	G189D	Deleterious	Probably damaging	Likely benign	Likely disease causing	Damaging	Deleterious	
13	D192G	Deleterious	Probably damaging	Likely deleterious	Likely disease causing	Damaging	Deleterious	
14	M236T	Deleterious	Probably damaging	Likely deleterious	Likely disease causing	Tolerated	Deleterious	
15	G237V	Deleterious	Probably damaging	Likely deleterious	Likely disease causing	Tolerated	Deleterious	
16	R254I	Deleterious	Probably damaging	Likely deleterious	Likely disease causing	Tolerated	Deleterious	
17	G274R	Deleterious	Probably damaging	Likely benign	Likely disease causing	Damaging	Deleterious	
18	G290C	Deleterious	Probably damaging	Likely deleterious	Likely disease causing	Damaging	Deleterious	
19	E316K	Deleterious	Probably damaging	Likely benign	Likely disease causing	Damaging	Deleterious	
20	P330L	Deleterious	Probably damaging	Likely benign	Likely disease causing	Damaging	Deleterious	
21	R333W	Deleterious	Probably damaging	Likely benign	Likely disease causing	Damaging	Deleterious	
22	R333Q	Deleterious	Possibly damaging	Likely benign	Likely disease causing	Damaging	Deleterious	

**Table 2:** Occurrence—in various types of tumors—of Polβ mutations causing an amino acid substitution at the same position as do the predicted SNPs of Polβ (see Table 1).

	SNP	Cbioportal https://www.cbioportal.org/ (accessed on 21 February 2021)	HiveBiochemistry https://hive.biochemistry.gwu.edu/biomuta (accessed on 21 February 2021)	COSMIC https://cancer.sanger.ac.uk/cosmic (accessed on 21 February 2021)
1	L22P			Carcinoma: L22F
2	R152L	Rectal adenocarcinoma: R152C	Lung cancer: R152P, malignant glioma: R152H	Lung cancer: R152P
3	G189D		Liver cancer: G189V	
4	D192G	Colon adenocarcinoma: D192H	Melanoma, colorectal cancer: D192H	
5	M236T		Liver cancer: M236I	
6	R254I	Uterine endometrioid carcinoma	Uterine cancer	
7	G274R	Melanoma: G274V		Malignant melanoma: G274V
8	G290C		Uterine cancer: G290D	
9	R333W		Prostate cancer	Prostate adenocarcinoma
10	R333Q	Colon adenocarcinoma	Colorectal cancer	

## Data Availability

Data are available upon request to O.A.K. Tel. +7 (383) 363-5174, E-mail: kladova@niboch.nsc.ru.

## Supplementary Materials

The following supporting information can be downloaded at: https://www.mdpi.com/article/10.3390/ijms23042390/s1.

## References

[1] Frederico L.A., Kunkel T.A., Shaw B.R. (1990). A sensitive genetic assay for the detection of cytosine deamination: Determination of rate constants and the activation energy. Biochemistry.

[2] Nakamura J., Walker V.E., Upton P.B., Chiang S.Y., Kow Y.W., Swenberg J.A. (1998). Highly sensitive apurinic/apyrimidinic site assay can detect spontaneous and chemically induced depurination under physiological conditions. Cancer Res..

[3] Burcham P.C. (1999). Internal hazards: Baseline DNA damage by endogenous products of normal metabolism. Mutat. Res..

[4] Wallace S.S. (2002). Biological consequences of free radical-damaged DNA bases. Free Radic. Biol. Med..

[5] Boiteux S., Guillet M. (2004). Abasic sites in DNA: Repair and biological consequences in Saccharomyces cerevisiae. DNA Repair.

[6] Evans M.D., Dizdaroglu M., Cooke M.S. (2004). Oxidative DNA damage and disease: Induction, repair and significance. Mutat. Res..

[7] Coppede F., Migliore L. (2015). DNA damage in neurodegenerative diseases. Mutat. Res. Mol. Mech. Mutagen..

[8] Leandro G.S., Sykora P., Bohr V.A. (2015). The impact of base excision DNA repair in age-related neurodegenerative diseases. Mutat. Res. Mol. Mech. Mutagen..

[9] Pascucci B., Stucki M., Jónsson Z.O., Dogliotti E., Hübscher U. (1999). Long patch base excision repair with purified human proteins. DNA ligase I as patch size mediator for DNA polymerases delta and epsilon. J. Biol. Chem..

[10] Frosina G., Fortini P., Rossi O., Carrozzino F., Raspaglio G., Cox L.S., Lane D.P., Abbondandolo A., Dogliotti E. (1996). Two pathways for base excision repair in mammalian cells. J. Biol. Chem..

[11] Srivastava D.K., Vande Berg B.J., Prasad R., Molina J.T., Beard W.A., Tomkinson A.E., Wilson S.H. (1998). Mammalian abasic site base excision repair: Identification of the reaction sequence and rate-determining steps. J. Biol. Chem..

[12] Matsumoto Y., Kim K. (1995). Excision of deoxyribose phosphate residues by DNA polymerase beta during DNA repair. Science.

[13] Klungland A., Lindahl T. (1997). Second pathway for completion of human DNA base excision-repair: Reconstitution with purified proteins and requirement for DNase IV (FEN1). EMBO J..

[14] Sobol R.W., Horton J.K., Kühn R., Gu H., Singhal R.K., Prasad R., Rajewsky K., Wilson S.H. (1996). Requirement of mammalian DNA polymerase-β in base-excision repair. Nature.

[15] Schärer O.D., Nash H.M., Jiricny J., Laval J., Verdine G.L. (1998). Specific Binding of a Designed Pyrrolidine Abasic Site Analog to Multiple DNA Glycosylases. J. Biol. Chem..

[16] Hill J.W., Hazra T.K., Izumi T., Mitra S. (2001). Stimulation of human 8-oxoguanine-DNA glycosylase by AP-endonuclease: Potential coordination of the initial steps in base excision repair. Nucleic Acids Res..

[17] Petronzelli F., Riccio A., Markham G.D., Seeholzer S.H., Stoerker J., Genuardi M., Yeung A.T., Matsumoto Y., Bellacosa A. (2000). Biphasic Kinetics of the Human DNA Repair Protein MED1 (MBD4), a Mismatch-specific DNA N-Glycosylase. J. Biol. Chem..

[18] Waters T.R., Swann P.F. (1998). Kinetics of the Action of Thymine DNA Glycosylase. J. Biol. Chem..

[19] Kladova O.A.O.A., Bazlekowa-Karaban M., Baconnais S., Piétrement O., Ishchenko A.A.A.A., Matkarimov B.T.B.T., Iakovlev D.A.D.A., Vasenko A., Fedorova O.S.O.S., Le Cam E. (2018). The role of the N-terminal domain of human apurinic/apyrimidinic endonuclease 1, APE1, in DNA glycosylase stimulation. DNA Repair.

[20] Sidorenko V.S., Nevinsky G.A., Zharkov D.O. (2007). Mechanism of interaction between human 8-oxoguanine-DNA glycosylase and AP endonuclease. DNA Repair.

[21] Xia L., Zheng L., Lee H.W., Bates S.E., Federico L., Shen B., O’Connor T.R. (2005). Human 3-Methyladenine-DNA Glycosylase: Effect of Sequence Context on Excision, Association with PCNA, and Stimulation by AP Endonuclease. J. Mol. Biol..

[22] Waters T.R., Gallinari P., Jiricnyl J., Swann P.F. (1999). Human Thymine DNA Glycosylase Binds to Apurinic Sites in DNA but Is Displaced by Human Apurinic Endonuclease 1. J. Biol. Chem..

[23] Esadze A., Rodriguez G., Cravens S.L., Stivers J.T. (2017). AP-Endonuclease 1 Accelerates Turnover of Human 8-Oxoguanine DNA Glycosylase by Preventing Retrograde Binding to the Abasic-Site Product. Biochemistry.

[24] Wiederhold L., Leppard J.B., Kedar P., Karimi-Busheri F., Rasouli-Nia A., Weinfeld M., Tomkinson A.E., Izumi T., Prasad R., Wilson S.H. (2004). AP endonuclease-independent DNA base excision repair in human cells. Mol. Cell.

[25] Kubota Y., Nash R.A., Klungland A., Schär P., Barnes D.E., Lindahl T. (1996). Reconstitution of DNA base excision-repair with purified human proteins: Interaction between DNA polymerase β and the XRCC1 protein. EMBO J..

[26] Dianova I.I., Sleeth K.M., Allinson S.L., Parsons J.L., Breslin C., Caldecott K.W., Dianov G.L. (2004). XRCC1-DNA polymerase β interaction is required for efficient base excision repair. Nucleic Acids Res..

[27] Marintchev A., Robertson A., Dimitriadis E.K., Prasad R., Wilson S.H., Mullen G.P. (2000). Domain specific interaction in the XRCC1-DNA polymerase β complex. Nucleic Acids Res..

[28] Gryk M.R., Marintchev A., Maciejewski M.W., Robertson A., Wilson S.H., Mullen G.P. (2002). Mapping of the interaction interface of DNA polymerase β with XRCC1. Structure.

[29] Kedar P.S., Kim S.J., Robertson A., Hou E., Prasad R., Horton J.K., Wilson S.H. (2002). Direct interaction between mammalian DNA polymerase β and proliferating cell nuclear antigen. J. Biol. Chem..

[30] Nash R.A., Caldecott K.W., Barnes D.E., Lindahl T. (1997). XRCC1 Protein Interacts with One of Two Distinct Forms of DNA Ligase III. Biochemistry.

[31] Masson M., Niedergang C., Schreiber V., Muller S., Menissier-de Murcia J., de Murcia G. (1998). XRCC1 is specifically associated with poly(ADP-ribose) polymerase and negatively regulates its activity following DNA damage. Mol. Cell. Biol..

[32] Das A., Wiederhold L., Leppard J.B., Kedar P., Prasad R., Wang H., Boldogh I., Karimi-Busheri F., Weinfeld M., Tomkinson A.E. (2006). NEIL2-initiated, APE-independent repair of oxidized bases in DNA: Evidence for a repair complex in human cells. DNA Repair.

[33] Campalans A., Marsin S., Nakabeppu Y., O’Connor T.R., Boiteux S., Radicella J.P. (2005). XRCC1 interactions with multiple DNA glycosylases: A model for its recruitment to base excision repair. DNA Repair.

[34] Akbari M., Solvang-Garten K., Hanssen-Bauer A., Lieske N.V., Pettersen H.S., Pettersen G.K., Wilson D.M., Krokan H.E., Otterlei M. (2010). Direct interaction between XRCC1 and UNG2 facilitates rapid repair of uracil in DNA by XRCC1 complexes. DNA Repair.

[35] Bennett R.A.O., Wilson D.M., Wong D., Demple B. (1997). Interaction of human apurinic endonuclease and DNA polymerase beta in the base excision repair pathway. Proc. Natl. Acad. Sci. USA.

[36] Moor N.A., Vasil’eva I.A., Anarbaev R.O., Antson A.A., Lavrik O.I. (2015). Quantitative characterization of protein-protein complexes involved in base excision DNA repair. Nucleic Acids Res..

[37] Lavrik O.I., Prasad R., Sobol R.W., Horton J.K., Ackerman E.J., Wilson S.H. (2001). Photoaffinity labeling of mouse fibroblast enzymes by a base excision repair intermediate: Evidence for the role of poly(ADP-ribose) polymerase-1 in DNA repair. J. Biol. Chem..

[38] Zhou T., Pan F., Cao Y., Han Y., Zhao J., Sun H., Zhou X., Wu X., He L., Hu Z. (2016). R152C DNA Pol β mutation impairs base excision repair and induces cellular transformation. Oncotarget.

[39] Pan F., Zhao J., Zhou T., Kuang Z., Dai H., Wu H., Sun H., Zhou X., Wu X., Hu Z. (2016). Mutation of DNA Polymerase β R137Q Results in Retarded Embryo Development Due to Impaired DNA Base Excision Repair in Mice. Sci. Rep..

[40] Nemec A.A., Abriola L., Merkel J.S., De Stanchina E., DeVeaux M., Zelterman D., Glazer P.M., Sweasy J.B. (2017). DNA polymerase beta germline variant confers cellular response to cisplatin therapy. Mol. Cancer Res..

[41] Almeida K.H., Sobol R.W. (2007). A unified view of base excision repair: Lesion-dependent protein complexes regulated by post-translational modification. DNA Repair.

[42] Fotiadou P., Henegariu O., Sweasy J.B. (2004). DNA polymerase β interacts with TRF2 and induces telomere dysfunction in a murine mammary cell line. Cancer Res..

[43] Muftuoglu M., Wong H.K., Imam S.Z., Wilson D.M., Bohr V.A., Opresko P.L. (2006). Telomere repeat binding factor 2 interacts with base excision repair proteins and stimulates DNA synthesis by DNA polymerase β. Cancer Res..

[44] Kidane D., Jonason A.S., Gorton T.S., Mihaylov I., Pan J., Keeney S., De Rooij D.G., Ashley T., Keh A., Liu Y. (2010). DNA polymerase Β is critical for mouse meiotic synapsis. EMBO J..

[45] Horton J.K., Srivastava D.K., Zmudzka B.Z., Wilson S.H. (1995). Strategic down-regulation of DNA polymerase β by antisense RNA sensitizes mammalian cells to specific DNA damaging agents. Nucleic Acids Res..

[46] Ray S., Breuer G., DeVeaux M., Zelterman D., Bindra R., Sweasy J.B. (2018). DNA polymerase beta participates in DNA end-joining. Nucleic Acids Res..

[47] Burgers P.M.J., Koonin E.V., Bruford E., Blanco L., Burtis K.C., Christman M.F., Copeland W.C., Friedberg E.C., Hanaoka F., Hinkle D.C. (2001). Eukaryotic DNA Polymerases: Proposal for a Revised Nomenclature. J. Biol. Chem..

[48] Wang T.S.F., Korn D. (1982). Specificity of the Catalytic Interaction of Human DNA Polymerase β with Nucleic Acid Substrates. Biochemistry.

[49] Prasad R., Beard W.A., Wilson S.H. (1994). Studies of gapped DNA substrate binding by mammalian DNA polymerase β. Dependence on 5′-phosphate group. J. Biol. Chem..

[50] Shu-Fong Wang T., Korn D. (1980). Reactivity of KB Cell Deoxyribonucleic Acid Polymerases α and β with Nicked and Gapped Deoxyribonucleic Acid. Biochemistry.

[51] Freemont P.S., Ollis D.L., Steitz T.A., Joyce C.M. (1986). A domain of the klenow fragment of Escherichia coli DNA polymerase I has polymerase but no exonuclease activity. Proteins Struct. Funct. Bioinform..

[52] Steitz T.A. (1993). DNA- and RNA-dependent DNA polymerases. Curr. Opin. Struct. Biol..

[53] Pelletier H., Sawaya M.R., Kumar A., Wilson S.H., Kraut J. (1994). Structures of Ternary Complexes of Rat DNA Polymerase, a DNA Template-Primer, and ddCTP. Science.

[54] Arndt J.W., Gong W., Zhong X., Showalter A.K., Liu J., Dunlap C.A., Lin Z., Paxson C., Tsai M.-D., Chan M.K. (2001). Insight into the Catalytic Mechanism of DNA Polymerase : Structures of Intermediate Complexes ^†,‡^. The coordinates have been deposited in the Protein Data Bank. PDB ID: Pol-DNA-Cr(III)‚dTMPPCP, 1huo; Pol-DNA-Cr(III). Biochemistry.

[55] Pelletier H., Sawaya M.R., Wolfle W., Wilson S.H., Kraut J. (1996). Crystal structures of human DNA polymerase β complexed with DNA: Implications for catalytic mechanism, processivity, and fidelity. Biochemistry.

[56] Bose-Basu B., DeRose E.F., Kirby T.W., Mueller G.A., Beard W.A., Wilson S.H., London R.E. (2004). Dynamic characterization of a DNA repair enzyme: NMR studies of [methyl-13C]methionine-labeled DNA polymerase β. Biochemistry.

[57] Joyce C.M., Steitz T.A. (1995). MINIREVIEW Polymerase Structures and Function: Variations on a Theme?. J. Bacteriol..

[58] Nakamura T., Zhao Y., Yamagata Y., Hua Y.J., Yang W. (2012). Watching DNA polymerase η make a phosphodiester bond. Nature.

[59] Whitaker A.M., Smith M.R., Schaich M.A., Freudenthal B.D. (2017). Capturing a mammalian DNA polymerase extending from an oxidized nucleotide. Nucleic Acids Res..

[60] Reed A.J., Suo Z. (2017). Time-Dependent Extension from an 8-Oxoguanine Lesion by Human DNA Polymerase Beta. J. Am. Chem. Soc..

[61] Reed A.J., Vyas R., Raper A.T., Suo Z. (2017). Structural insights into the post-chemistry steps of nucleotide incorporation catalyzed by a DNA polymerase. J. Am. Chem. Soc..

[62] Vyas R., Reed A.J., Tokarsky E.J., Suo Z. (2015). Viewing Human DNA Polymerase β Faithfully and Unfaithfully Bypass an Oxidative Lesion by Time-Dependent Crystallography. J. Am. Chem. Soc..

[63] Freudenthal B.D., Beard W.A., Perera L., Shock D.D., Kim T., Schlick T., Wilson S.H. (2015). Uncovering the polymerase-induced cytotoxicity of an oxidized nucleotide. Nature.

[64] Freudenthal B.D., Beard W.A., Shock D.D., Wilson S.H. (2013). Observing a DNA polymerase choose right from wrong. Cell.

[65] Gao Y., Yang W. (2016). Capture of A Third Mg^2+^ is Essential for Catalyzing DNA Synthesis. Science.

[66] Raper A.T., Reed A.J., Suo Z. (2018). Kinetic Mechanism of DNA Polymerases: Contributions of Conformational Dynamics and a Third Divalent Metal Ion. Chem. Rev..

[67] Yang W., Weng P.J., Gao Y. (2016). A new paradigm of DNA synthesis: Three-metal-ion catalysis. Cell Biosci..

[68] Jamsen J.A., Beard W.A., Pedersen L.C., Shock D.D., Moon A.F., Krahn J.M., Bebenek K., Kunkel T.A., Wilson S.H. (2017). Time-lapse crystallography snapshots of a double-strand break repair polymerase in action. Nat. Commun..

[69] Singhal R.K., Wilson S.H. (1993). Short gap-filling synthesis by DNA polymerase β is processive. J. Biol. Chem..

[70] Nowak R., Kulik J., Siedlecki J.A. (1987). The ability of DNA polymerase beta to synthesize DNA beyond the gap with displacement of the non-replicated strand. Acta. Biochim. Pol..

[71] Prasad R., Dianov G.L., Bohr V.A., Wilson S.H. (2000). FEN1 stimulation of DNA polymerase β mediates an excision step in mammalian long patch base excision repair. J. Biol. Chem..

[72] Prasad R., Lavrik O.I., Kim S.J., Kedar P., Yang X.P., Vande Berg B.J., Wilson S.H. (2001). DNA polymerase β-mediated long patch base excision repair: Poly(ADP-ribose) polymerase-1 stimulates strand displacement DNA synthesis. J. Biol. Chem..

[73] Sukhanova M.V., Khodyreva S.N., Lebedeva N.A., Prasad R., Wilson S.H., Lavrik O.I. (2005). Human base excision repair enzymes apurinic/apyrimidinic endonuclease1 (APE1), DNA polymerase β and poly(ADP-ribose) polymerase 1: Interplay between strand-displacement DNA synthesis and proofreading exonuclease activity. Nucleic Acids Res..

[74] Prasad R., Beard W.A., Strauss P.R., Wilson S.H. (1998). Human DNA polymerase β deoxyribose phosphate lyase: Substrate specificity and catalytic mechanism. J. Biol. Chem..

[75] Matsumoto Y., Kim K., Katz D.S., Feng J.A. (1998). Catalytic center of DNA polymerase β for excision of deoxyribose phosphate groups. Biochemistry.

[76] Xu G., Herzig M., Rotrekl V., Walter C.A. (2008). Base excision repair, aging and health span. Mech. Ageing Dev..

[77] Starcevic D., Dalal S., Sweasy J.B. (2004). Is there a link between DNA polymerase β and cancer?. Cell Cycle.

[78] Copani A., Hoozemans J.J.M., Caraci F., Calafiore M., Van Haastert E.S., Veerhuis R., Rozemuller A.J.M., Aronica E., Sortino M.A., Nicoletti F. (2006). DNA polymerase-β is expressed early in neurons of Alzheimer’s disease brain and is loaded into DNA replication forks in neurons challenged with β-amyloid. J. Neurosci..

[79] Copani A., Caraci F., Hoozemans J.J.M., Calafiore M., Angela Sortino M., Nicoletti F. (2007). The nature of the cell cycle in neurons: Focus on a “non-canonical” pathway of DNA replication causally related to death. Biochim. Biophys. Acta. Mol. Basis Dis..

[80] Loeb L.A., Springgate C.F., Battula N. (1974). Errors in DNA Replication as a Basis of Malignant Changes. Cancer Res..

[81] Loeb L.A. (2016). Human cancers express a mutator phenotype: Hypothesis, origin, and consequences. Cancer Res..

[82] Bronner C.E., Baker S.M., Morrison P.T., Warren G., Smith L.G., Lescoe M.K., Kane M., Earabino C., Lipford J., Lindblom A. (1994). Mutation in the DNA mismatch repair gene homologue hMLH 1 is associated with hereditary non-polyposis colon cancer. Nature.

[83] Kothandapani A., Sawant A., Dangeti V.S.M.N., Sobol R.W., Patrick S.M. (2013). Epistatic role of base excision repair and mismatch repair pathways in mediating cisplatin cytotoxicity. Nucleic Acids Res..

[84] Lang T., Dalal S., Chikova A., DiMaio D., Sweasy J.B. (2007). The E295K DNA Polymerase Beta Gastric Cancer-Associated Variant Interferes with Base Excision Repair and Induces Cellular Transformation. Mol. Cell. Biol..

[85] Bhattacharyya N., Chen H.-C., Comhair S., Erzurum S.C., Banerjee S. (1999). Variant Forms of DNA Polymerase beta in Primary Lung Carcinomas. DNA Cell Biol..

[86] Opresko P.L., Sweasy J.B., Eckert K.A. (1998). The mutator form of polymerase β with amino acid substitution at tyrosine 265 in the hinge region displays an increase in both base substitution and frame shift errors. Biochemistry.

[87] Starcevic D., Dalal S., Sweasy J. (2005). Hinge residue Ile260 of DNA polymerase β is important for enzyme activity and fidelity. Biochemistry.

[88] Collins F., Brooks L., Chakravarti A. (1998). A DNA polymorphism discovery resource for research on human genetic variation. Genome Res..

[89] Donigan K.A., Hile S.E., Eckert K.A., Sweasy J.B. (2012). The human gastric cancer-associated DNA polymerase β variant D160N is a mutator that induces cellular transformation. DNA Repair.

[90] Hall J., Marcel V., Bolin C., Fernet M., Tartier L., Vaslin L., Hainaut P. (2009). The associations of sequence variants in DNA-repair and cell-cycle genes with cancer risk: Genotype-phenotype correlations. Biochem. Soc. Trans..

[91] Nemec A.A., Donigan K.A., Murphy D.L., Jaegers J., Sweasy J.B. (2012). Colon cancer-associated DNA polymerase β variant induces genomic instability and cellular transformation. J. Biol. Chem..

[92] Nemec A.A., Murphy D.L., Donigan K.A., Sweasy J.B. (2014). The S229L colon tumor-associated variant of DNA polymerase β induces cellular transformation as a result of decreased polymerization efficiency. J. Biol. Chem..

[93] Ng P.C., Henikoff S. (2002). Accounting for Human Polymorphisms Predicted to Affect Protein Function. Genome Res..

[94] Ng P.C., Henikoff S. (2001). Predicting Deleterious Amino Acid Substitutions. Genome Res..

[95] Adzhubei I., Schmidt S., Peshkin L., Ramensky V., Gerasimova A., Bork P., Kondrashov A., Sunyaev S. (2010). A method and server for predicting damaging missense mutations. Nat. Methods.

[96] Martin K., Daniela M.W., Preti J., Brain J.O., Gregory M.C., Jay S. (2014). A general framework for estimating the relative pathogenicity of human genetic variants. Nat. Genet..

[97] Ioannidis N.M., Rothstein J.H., Pejaver V., Middha S., McDonnell S.K., Baheti S., Musolf A., Li Q., Holzinger E., Karyadi D. (2016). REVEL: An Ensemble Method for Predicting the Pathogenicity of Rare Missense Variants. Am. J. Hum. Genet..

[98] Dong C., Wei P., Jian X., Gibbs R., Boerwinkle E., Wang K., Liu X. (2015). Comparison and integration of deleteriousness prediction methods for nonsynonymous SNVs in whole exome sequencing studies. Hum. Mol. Genet..

[99] Sato Y., Yoshizato T., Shiraishi Y., Maekawa S., Okuno Y., Kamura T., Shimamura T., Sato-Otsubo A., Nagae G., Suzuki H. (2013). Integrated molecular analysis of clear-cell renal cell carcinoma. Nat. Genet..

[100] Kirby T.W., Derose E.F., Beard W.A., Shock D.D., Wilson S.H., London R.E. (2014). Substrate rescue of DNA polymerase β containing a catastrophic L22P mutation. Biochemistry.

[101] Rozacky J., Nemec A.A., Sweasy J.B., Kidane D. (2015). Gastric cancer associated variant of DNA polymerase beta (Leu22Pro) promotes DNA replication associated double strand breaks. Oncotarget.

[102] Dalal S., Chikova A., Jaeger J., Sweasy J.B. (2008). The Leu22Pro tumor-associated variant of DNA polymerase beta is dRP lyase deficient. Nucleic Acids Res..

[103] El-Andaloussi N., Valovka T., Toueille M., Steinacher R., Focke F., Gehrig P., Covic M., Hassa P.O., Schär P., Hübscher U. (2006). Arginine Methylation Regulates DNA Polymerase β. Mol. Cell.

[104] Gu H., Marth J.D., Orban P.C., Mossmann H., Rajewsky K. (1994). Deletion of a DNA Polymerase β Gene Segment in T Cells Using Cell Type-Specific Gene Targeting. Science.

[105] Poltoratsky V., Horton J.K., Prasad R., Wilson S.H. (2005). REV1 mediated mutagenesis in base excision repair deficient mouse fibroblast. DNA Repair.

[106] Albertella M.R., Lau A., O’Connor M.J. (2005). The overexpression of specialized DNA polymerases in cancer. DNA Repair.

[107] Yang J., Parsons J., Nicolay N.H., Caporali S., Harrington C.F., Singh R., Finch D., Datri S., Farmer P.B., Johnston P.G. (2009). Cells deficient in the base excision repair protein, DNA polymerase beta, are hypersensitive to oxaliplatin chemotherapy. Oncogene.

[108] Canitrot Y., Cazaux C., Fréchet M., Bouayadi K., Lesca C., Salles B., Hoffmann J.S. (1998). Overexpression of DNA polymerase β in cell results in a mutator phenotype and a decreased sensitivity to anticancer drugs. Proc. Natl. Acad. Sci. USA.

[109] Nicolay N.H., Helleday T., Sharma R.A. (2011). Biological Relevance of DNA Polymerase Beta and Translesion Synthesis Polymerases to Cancer and its Treatment. Curr. Mol. Pharmacol..

[110] Yamtich J., Starcevic D., Lauper J., Smith E., Shi I., Rangarajan S., Jaeger J., Sweasy J.B. (2010). Hinge residue I174 is critical for proper dNTP selection by DNA polymerase beta. Biochemistry.

[111] Yamtich J., Nemec A.A., Keh A., Sweasy J.B. (2012). A Germline Polymorphism of DNA Polymerase Beta Induces Genomic Instability and Cellular Transformation. PLoS Genet..

[112] Iwanaga A., Ouchida M., Miyazaki K., Hori K., Mukai T. (1999). Functional mutation of DNA polymerase β found in human gastric cancer-Inability of the base excision repair in vitro. Mutat. Res. DNA Repair.

[113] Sawaya M.R., Pelletier H., Kumar A., Wilson S.H., Kraut J. (1994). Crystal structure of rat DNA polymerase β: Evidence for a common polymerase mechanism. Science.

[114] Prasad R., Batra V.K., Yang X.P., Krahn J.M., Pedersen L.C., Beard W.A., Wilson S.H. (2005). Structural insight into the DNA polymerase β deoxyribose phosphate lyase mechanism. DNA Repair.

[115] Lyu P.C., Sherman J.C., Chen A., Kallenbach N.R. (1991). α-Helix stabilization by natural and unnatural amino acids with alkyl side chains. Proc. Natl. Acad. Sci. USA.

[116] Murphy D.L., Donigan K.A., Jaeger J., Sweasy J.B. (2012). The E288K colon tumor variant of DNA polymerase β is a sequence specific mutator. Biochemistry.

[117] Chan K., Houlbrook S., Zhang Q.M., Harrison M., Hickson I.D., Dianov G.L. (2007). Overexpression of DNA polymerase β results in an increased rate of frameshift mutations during base excision repair. Mutagenesis.

[118] Guo Z., Zheng L., Dai H., Zhou M., Xu H., Shen B. (2009). Human DNA polymerase β polymorphism, Arg137Gln, impairs its polymerase activity and interaction with PCNA and the cellular base excision repair capacity. Nucleic Acids Res..

[119] Wang M., Li E., Lin L., Kumar A.K., Pan F., He L., Zhang J., Hu Z., Guo Z. (2019). Enhanced activity of variant DNA polymerase b (D160G) contributes to cisplatin therapy by impeding the efficiency of NER. Mol. Cancer Res..

[120] Alnajjar K.S., Garcia-Barboza B., Negahbani A., Nakhjiri M., Kashemirov B., McKenna C., Goodman M.F., Sweasy J.B. (2017). A change in the rate-determining step of polymerization by the K289M DNA polymerase β cancer-associated variant. Biochemistry.

[121] Lang T., Maitra M., Starcevic D., Li S.-X., Sweasy J.B. (2004). A DNA polymerase mutant from colon cancer cells induces mutations. Proc. Natl. Acad. Sci. USA.

[122] Murphy D.L., Kosa J., Jaeger J., Sweasy J.B. (2008). The Asp285 variant of DNA polymerase beta extends mispaired primer termini via increased nucleotide binding. Biochemistry.

