# Supplementary material for: The Role of Natural Polymorphic Variants of DNA Polymerase β in DNA Repair
Source: Int J Mol Sci. 2022 Feb 21;23(4):2390. doi: 10.3390/ijms23042390 (PMC8877055; doi:10.3390/ijms23042390)
Supplement: Supplementary file 1 [file ijms-23-02390-s001.zip › ijms-1595447-supplementary.pdf]

Supplementary Materials

The Role of Natural Polymorphic Variants of DNA Polymerase β in DNA Repair

Olga A. Kladova [1,*], Olga S. Fedorova [1] and Nikita A. Kuznetsov [1,2,*]

[1] Institute of Chemical Biology and Fundamental Medicine, Siberian Branch of Russian Academy of Sciences, 630090 Novosibirsk, Russia; fedorova@niboch.nsc.ru

[2] Department of Natural Sciences, Novosibirsk State University, 630090 Novosibirsk, Russia

* Correspondence: kladova@niboch.nsc.ru (O.A.K.); nikita.kuznetsov@niboch.nsc.ru (N.A.K.)

Table S1. Analysis of known missense SNPs of Polβ. The green lines (neutral) indicate no probability of damaging effect of mutation, as none of the software packages predicted negative consequences of amino acid substitution. The yellow lines indicate low probability, as the effect was predicted in 1 or 2 programs. The orange lines indicate medium probability as the effect was predicted in 3 or 4 programs. The red lines indicate high probability as 5 or 6 of the tested prediction software showed damaging effect of mutation.

1	SNP	SIFT	PolyPhen	CADD	Revel	Metalr	Provean	Result
2	A6P	Deleterious	Possibly damaging	Likely benign	Likely benign	Tolerated	Deleterious	Medium
3	P7S	Tolerated	Benign	Likely benign	Likely benign	Tolerated	Neutral	Neutral
4	P7Q	Deleterious	Possibly damaging	Likely benign	Likely benign	Tolerated	Deleterious	Medium
5	Q8R	Tolerated	Benign	Likely benign	Likely benign	Tolerated	Neutral	Neutral
6	E9D	Tolerated	Benign	Likely benign	Likely benign	Tolerated	Neutral	Neutral
7	L11F	Tolerated	Benign	Likely benign	Likely benign	Tolerated	Neutral	Neutral
8	L11P	Tolerated	Benign	Likely benign	Likely benign	Tolerated	Neutral	Neutral
9	G13R	Tolerated	Benign	Likely benign	Likely benign	Tolerated	Neutral	Neutral
10	G13W	Deleterious	Possibly damaging	Likely benign	Likely benign	Tolerated	Neutral	Low
11	G13E	Tolerated	Benign	Likely benign	Likely benign	Tolerated	Neutral	Neutral
12	M18R	Deleterious	Benign	Likely benign	Likely benign	Tolerated	Deleterious	Low
13	L19P	Deleterious	Probably damaging	Likely deleterious	Likely disease causing	Damaging	Deleterious	High
14	L22P	Deleterious	Probably damaging	Likely deleterious	Likely disease causing	Tolerated	Deleterious	High
15	A23S	Tolerated	Possibly damaging	Likely benign	Likely benign	Tolerated	Neutral	Low
16	N24H	Deleterious	Possibly damaging	Likely benign	Likely benign	Tolerated	Neutral	Low
17	N24D	Tolerated	Benign	Likely benign	Likely benign	Tolerated	Neutral	Neutral
18	N28K	Deleterious	Probably damaging	Likely benign	Likely benign	Tolerated	Deleterious	Medium
19	V29M	Deleterious	Probably damaging	Likely deleterious	Likely benign	Tolerated	Neutral	Medium
20	S30N	Tolerated	Benign	Likely benign	Likely benign	Tolerated	Neutral	Neutral
21	A32T	Tolerated	Benign	Likely benign	Likely benign	Tolerated	Neutral	Neutral
22	A32S	Tolerated	Benign	Likely benign	Likely benign	Tolerated	Neutral	Neutral

23	A32V	Tolerated	Benign	Likely benign	Likely benign	Tolerated	Neutral	Neutral
24	I33V	Tolerated	Benign	Likely benign	Likely benign	Tolerated	Neutral	Neutral
25	K35E	Deleterious	Probably damaging	Likely deleterious	Likely disease causing	Tolerated	Deleterious	High
26	N37S	Tolerated	Benign	Likely benign	Likely benign	Tolerated	Deleterious	Low
27	A38V	Tolerated	Benign	Likely benign	Likely benign	Tolerated	Deleterious	Low
28	R40G	Deleterious	Benign	Likely benign	Likely benign	Tolerated	Deleterious	Low
29	R40K	Tolerated	Benign	Likely deleterious	Likely benign	Tolerated	Neutral	Low
30	A42T	Deleterious	Probably damaging	Likely benign	Likely disease causing	Damaging	Deleterious	High
31	A43T	Deleterious	Possibly damaging	Likely benign	Likely benign	Tolerated	Deleterious	Medium
32	S44Y	Deleterious	Benign	Likely benign	Likely benign	Tolerated	Deleterious	Low
33	I46T	Deleterious	Possibly damaging	Likely benign	Likely disease causing	Tolerated	Deleterious	Medium
34	K48R	Tolerated	Benign	Likely benign	Likely benign	Tolerated	Neutral	Neutral
35	P50S	Tolerated	Benign	Likely benign	Likely benign	Tolerated	Deleterious	Low
36	H51Y	Tolerated	Benign	Likely benign	Likely benign	Tolerated	Neutral	Neutral
37	K52E	Tolerated	Benign	Likely benign	Likely benign	Tolerated	Neutral	Neutral
38	K54T	Tolerated	Benign	Likely benign	Likely benign	Tolerated	Neutral	Neutral
39	G56R	Deleterious	Probably damaging	Likely benign	Likely benign	Tolerated	Deleterious	Medium
40	K61T	Deleterious	Possibly damaging	Likely benign	Likely benign	Tolerated	Deleterious	Medium
41	P63T	Deleterious	Benign	Likely benign	Likely benign	Tolerated	Deleterious	Low
42	V65L	Deleterious	Benign	Likely benign	Likely benign	Tolerated	Neutral	Low
43	G66R	Deleterious	Probably damaging	Likely benign	Likely disease causing	Damaging	Deleterious	High
44	T67I	Tolerated	Benign	Likely benign	Likely benign	Tolerated	Neutral	Neutral
45	E71G	Deleterious	Benign	Likely benign	Likely benign	Tolerated	Deleterious	Low
46	I73V	Tolerated	Benign	Likely benign	Likely benign	Tolerated	Neutral	Neutral
47	D74G	Tolerated	Benign	Likely benign	Likely benign	Tolerated	Deleterious	Low
48	R83C	Deleterious	Possibly damaging	Likely deleterious	Likely benign	Tolerated	Deleterious	Medium

49	R83H	Deleterious	Benign	Likely benign	Likely benign	Tolerated	Neutral	Low
50	K87E	Tolerated	Benign	Likely benign	Likely benign	Tolerated	Neutral	Neutral
51	K87N	Tolerated	Benign	Likely deleterious	Likely benign	Tolerated	Neutral	Low
52	I88V	Tolerated	Benign	Likely benign	Likely benign	Tolerated	Neutral	Neutral
53	R89W	Deleterious	Probably damaging	Likely deleterious	Likely benign	Tolerated	Deleterious	Medium
54	R89Q	Tolerated	Benign	Likely benign	Likely benign	Tolerated	Neutral	Neutral
55	Q90K	Tolerated	Benign	Likely benign	Likely benign	Tolerated	Neutral	Neutral
56	Q90R	Tolerated	Benign	Likely benign	Likely benign	Tolerated	Neutral	Neutral
57	Q90H	Deleterious	Benign	Likely benign	Likely benign	Tolerated	Neutral	Low
58	D91G	Tolerated	Possibly damaging	Likely deleterious	Likely benign	Tolerated	Deleterious	Medium
59	T93M	Deleterious	Benign	Likely benign	Likely benign	Tolerated	Deleterious	Low
60	S96F	Deleterious	Possibly damaging	Likely benign	Likely benign	Tolerated	Deleterious	Medium
61	N98S	Tolerated	Benign	Likely benign	Likely benign	Tolerated	Neutral	Neutral
62	F99L	Tolerated	Benign	Likely benign	Likely benign	Tolerated	Neutral	Neutral
63	T101N	Deleterious	Possibly damaging	Likely benign	Likely benign	Tolerated	Deleterious	Medium
64	R102Q	Tolerated	Benign	Likely benign	Likely benign	Tolerated	Neutral	Neutral
65	P108Q	Deleterious	Probably damaging	Likely benign	Likely benign	Tolerated	Deleterious	Medium
66	A110T	Tolerated	Benign	Likely benign	Likely benign	Tolerated	Neutral	Neutral
67	F114L	Tolerated	Benign	Likely benign	Likely benign	Tolerated	Neutral	Neutral
68	G118V	Deleterious	Probably damaging	Likely deleterious	Likely disease causing	Damaging	Deleterious	High
69	I119V	Tolerated	Benign	Likely benign	Likely benign	Tolerated	Neutral	Neutral
70	L122R	Deleterious	Probably damaging	Likely benign	Likely disease causing	Damaging	Deleterious	High
71	E123D	Tolerated	Benign	Likely benign	Likely benign	Tolerated	Neutral	Neutral
72	R126K	Tolerated	Benign	Likely benign	Likely benign	Tolerated	Neutral	Neutral
73	K127R	Deleterious	Benign	Likely benign	Likely benign	Tolerated	Neutral	Low
74	N128H	Tolerated	Benign	Likely benign	Likely benign	Tolerated	Neutral	Neutral

75	N128D	Tolerated	Benign	Likely benign	Likely benign	Tolerated	Neutral	Neutral
76	E129G	Deleterious	Benign	Likely benign	Likely benign	Tolerated	Deleterious	Low
77	E129D	Tolerated	Benign	Likely benign	Likely benign	Tolerated	Neutral	Neutral
78	D130N	Tolerated	Benign	Likely benign	Likely benign	Tolerated	Neutral	Neutral
79	D130G	Tolerated	Benign	Likely benign	Likely benign	Tolerated	Deleterious	Low
80	D130E	Tolerated	Benign	Likely benign	Likely benign	Tolerated	Neutral	Neutral
81	N133K	Deleterious	Possibly damaging	Likely benign	Likely benign	Tolerated	Deleterious	Medium
82	H134R	Tolerated	Benign	Likely benign	Likely benign	Tolerated	Deleterious	Low
83	R137Q	Tolerated	Benign	Likely benign	Likely benign	Tolerated	Neutral	Neutral
84	R137P	Tolerated	Possibly damaging	Likely benign	Likely benign	Tolerated	Deleterious	Medium
85	Y142H	Tolerated	Possibly damaging	Likely benign	Likely benign	Tolerated	Neutral	Low
86	G144A	Tolerated	Benign	Likely benign	Likely benign	Tolerated	Neutral	Neutral
87	R149G	Deleterious	Benign	Likely benign	Likely benign	Tolerated	Deleterious	Medium
88	R149I	Deleterious	Possibly damaging	Likely deleterious	Likely disease causing	Tolerated	Deleterious	High
89	I150T	Deleterious	Probably damaging	Likely benign	Likely disease causing	Tolerated	Deleterious	Medium
90	P151L	Deleterious	Possibly damaging	Likely benign	Likely disease causing	Damaging	Deleterious	High
91	R152H	Deleterious	Probably damaging	Likely deleterious	Likely benign	Tolerated	Deleterious	Medium
92	R152L	Deleterious	Probably damaging	Likely deleterious	Likely benign	Damaging	Deleterious	High
93	E154A	Deleterious	Probably damaging	Likely deleterious	Likely disease causing	Damaging	Deleterious	High
94	M155L	Tolerated	Benign	Likely benign	Likely benign	Tolerated	Neutral	Neutral
95	M155I	Tolerated	Benign	Likely benign	Likely benign	Tolerated	Neutral	Neutral
96	Q157E	Tolerated	Benign	Likely benign	Likely benign	Tolerated	Neutral	Neutral
97	Q159E	Tolerated	Benign	Likely benign	Likely benign	Tolerated	Neutral	Neutral
98	D160G	Tolerated	Benign	Likely benign	Likely benign	Tolerated	Neutral	Neutral
99	I161V	Tolerated	Benign	Likely benign	Likely benign	Tolerated	Neutral	Neutral
100	V162I	Tolerated	Benign	Likely benign	Likely benign	Tolerated	Neutral	Neutral

101	N164D	Tolerated	Benign	Likely benign	Likely benign	Tolerated	Neutral	Neutral
102	N164S	Tolerated	Benign	Likely benign	Likely benign	Tolerated	Neutral	Neutral
103	E165K	Tolerated	Benign	Likely benign	Likely benign	Tolerated	Neutral	Neutral
104	E165D	Tolerated	Benign	Likely benign	Likely benign	Tolerated	Neutral	Neutral
105	K167E	Tolerated	Benign	Likely benign	Likely benign	Tolerated	Neutral	Neutral
106	V169M	Deleterious	Benign	Likely benign	Likely benign	Tolerated	Neutral	Low
107	D170N	Tolerated	Benign	Likely benign	Likely benign	Tolerated	Deleterious	Low
108	D170V	Deleterious	Possibly damaging	Likely benign	Likely benign	Tolerated	Deleterious	Medium
109	S171A	Tolerated	Benign	Likely benign	Likely benign	Tolerated	Neutral	Neutral
110	Y173C	Deleterious	Benign	Likely benign	Likely benign	Tolerated	Deleterious	Medium
111	I174V	Tolerated	Benign	Likely benign	Likely benign	Tolerated	Neutral	Neutral
112	A175T	Tolerated	Benign	Likely benign	Likely benign	Tolerated	Neutral	Neutral
113	T176A	Tolerated	Benign	Likely benign	Likely benign	Tolerated	Neutral	Neutral
114	V177L	Tolerated	Benign	Likely benign	Likely benign	Tolerated	Neutral	Neutral
115	S180R	Deleterious	Probably damaging	Likely benign	Likely disease causing	Tolerated	Deleterious	Medium
116	R182G	Deleterious	Probably damaging	Likely benign	Likely disease causing	Tolerated	Deleterious	Medium
117	S187Y	Deleterious	Benign	Likely benign	Likely benign	Tolerated	Deleterious	Low
118	G189D	Deleterious	Probably damaging	Likely benign	Likely disease causing	Damaging	Deleterious	High
119	M191I	Tolerated	Benign	Likely benign	Likely benign	Tolerated	Neutral	Neutral
120	D192G	Deleterious	Probably damaging	Likely deleterious	Likely disease causing	Damaging	Deleterious	High
121	T196N	Deleterious	Possibly damaging	Likely benign	Likely benign	Tolerated	Deleterious	Medium
122	T196S	Tolerated	Benign	Likely benign	Likely benign	Tolerated	Neutral	Neutral
123	T196I	Deleterious	Benign	Likely benign	Likely benign	Tolerated	Deleterious	Low
124	F200L	Tolerated	Benign	Likely benign	Likely benign	Tolerated	Deleterious	Low
125	F200V	Tolerated	Benign	Likely benign	Likely benign	Tolerated	Deleterious	Low
126	F200S	Tolerated	Possibly damaging	Likely benign	Likely benign	Tolerated	Deleterious	Low

127	S202A	Deleterious	Benign	Likely benign	Likely benign	Tolerated	Neutral	Low
128	T205I	Tolerated	Benign	Likely benign	Likely benign	Tolerated	Neutral	Neutral
129	P208S	Tolerated	Benign	Likely benign	Likely benign	Tolerated	Neutral	Neutral
130	L210R	Deleterious	Probably damaging	Likely benign	Likely disease causing	Tolerated	Deleterious	Medium
131	H212R	Tolerated	Benign	Likely benign	Likely benign	Tolerated	Neutral	Neutral
132	Q213R	Tolerated	Benign	Likely benign	Likely benign	Tolerated	Neutral	Neutral
133	V215M	Deleterious	Possibly damaging	Likely benign	Likely benign	Tolerated	Neutral	Low
134	E216Q	Tolerated	Benign	Likely benign	Likely benign	Tolerated	Neutral	Neutral
135	Q217P	Tolerated	Benign	Likely benign	Likely benign	Tolerated	Neutral	Neutral
136	Q219R	Tolerated	Benign	Likely benign	Likely benign	Tolerated	Neutral	Neutral
137	K220N	Tolerated	Benign	Likely benign	Likely benign	Tolerated	Neutral	Neutral
138	I224V	Tolerated	Benign	Likely benign	Likely benign	Tolerated	Neutral	Neutral
139	D226H	Deleterious	Probably damaging	Likely deleterious	Likely benign	Tolerated	Deleterious	Medium
140	K230Q	Tolerated	Benign	Likely benign	Likely benign	Tolerated	Neutral	Neutral
141	M236V	Deleterious	Possibly damaging	Likely benign	Likely benign	Tolerated	Deleterious	Medium
142	M236T	Deleterious	Probably damaging	Likely deleterious	Likely disease causing	Tolerated	Deleterious	High
143	G237V	Deleterious	Probably damaging	Likely deleterious	Likely disease causing	Tolerated	Deleterious	High
144	P242S	Deleterious	Benign	Likely benign	Likely benign	Tolerated	Deleterious	Low
145	P242R	Deleterious	Benign	Likely benign	Likely benign	Tolerated	Deleterious	Low
146	N245D	Tolerated	Benign	Likely benign	Likely benign	Tolerated	Neutral	Neutral
147	D246G	Tolerated	Benign	Likely benign	Likely benign	Tolerated	Neutral	Neutral
148	K248E	Tolerated	Benign	Likely benign	Likely benign	Tolerated	Neutral	Neutral
149	E249K	Tolerated	Benign	Likely benign	Likely benign	Tolerated	Neutral	Neutral
150	Y250F	Tolerated	Benign	Likely benign	Likely benign	Tolerated	Neutral	Neutral
151	P251L	Tolerated	Benign	Likely benign	Likely benign	Tolerated	Neutral	Neutral
152	H252R	Deleterious	Benign	Likely benign	Likely benign	Tolerated	Deleterious	Low

153	R254I	Deleterious	Probably damaging	Likely deleterious	Likely disease causing	Tolerated	Deleterious	High
154	I255V	Tolerated	Benign	Likely benign	Likely benign	Tolerated	Neutral	Neutral
155	I255T	Deleterious	Probably damaging	Likely benign	Likely disease causing	Tolerated	Deleterious	Medium
156	I257V	Tolerated	Benign	Likely benign	Likely benign	Tolerated	Neutral	Low
157	I257S	Deleterious	Probably damaging	Likely benign	Likely disease causing	Tolerated	Deleterious	Medium
158	I257M	Deleterious	Possibly damaging	Likely benign	Likely benign	Tolerated	Neutral	Low
159	L259S	Deleterious	Probably damaging	Likely benign	Likely disease causing	Tolerated	Deleterious	Medium
160	I260L	Tolerated	Benign	Likely benign	Likely benign	Tolerated	Neutral	Neutral
161	I260M	Deleterious	Possibly damaging	Likely benign	Likely benign	Tolerated	Neutral	Low
162	P261L	Deleterious	Probably damaging	Likely benign	Likely disease causing	Tolerated	Deleterious	Medium
163	Y266C	Deleterious	Probably damaging	Likely benign	Likely benign	Tolerated	Deleterious	Medium
164	L270F	Deleterious	Probably damaging	Likely benign	Likely benign	Tolerated	Deleterious	Medium
165	Y271H	Tolerated	Possibly damaging	Likely benign	Likely disease causing	Tolerated	Deleterious	Medium
166	Y271C	Deleterious	Probably damaging	Likely benign	Likely disease causing	Tolerated	Deleterious	Medium
167	G274R	Deleterious	Probably damaging	Likely benign	Likely disease causing	Damaging	Deleterious	High
168	N279S	Deleterious	Probably damaging	Likely benign	Likely disease causing	Tolerated	Deleterious	Medium
169	M282V	Deleterious	Possibly damaging	Likely benign	Likely disease causing	Tolerated	Deleterious	Medium
170	R283G	Deleterious	Probably damaging	Likely benign	Likely disease causing	Tolerated	Deleterious	Medium
171	H285Y	Tolerated	Benign	Likely benign	Likely benign	Tolerated	Deleterious	Low
172	H285P	Tolerated	Possibly damaging	Likely benign	Likely benign	Tolerated	Deleterious	Low
173	K289R	Tolerated	Benign	Likely benign	Likely benign	Tolerated	Neutral	Neutral
174	G290C	Deleterious	Probably damaging	Likely deleterious	Likely disease causing	Damaging	Deleterious	High
175	G290D	Deleterious	Benign	Likely benign	Likely benign	Damaging	Deleterious	Medium
176	I293T	Deleterious	Benign	Likely benign	Likely benign	Tolerated	Deleterious	Low
177	N294I	Deleterious	Probably damaging	Likely benign	Likely disease causing	Tolerated	Deleterious	Medium
178	E295D	Deleterious	Probably damaging	Likely benign	Likely benign	Tolerated	Deleterious	Medium

179	R299C	Deleterious	Benign	Likely benign	Likely benign	Tolerated	Deleterious	Low
180	R299H	Deleterious	Possibly damaging	Likely benign	Likely benign	Tolerated	Deleterious	Medium
181	E309D	Tolerated	Benign	Likely benign	Likely benign	Tolerated	Neutral	Neutral
182	L311M	Deleterious	Possibly damaging	Likely benign	Likely benign	Tolerated	Neutral	Low
183	S315R	Deleterious	Possibly damaging	Likely benign	Likely benign	Tolerated	Deleterious	Medium
184	E316K	Deleterious	Probably damaging	Likely benign	Likely disease causing	Damaging	Deleterious	High
185	K317N	Deleterious	Possibly damaging	Likely benign	Likely benign	Tolerated	Neutral	Low
186	D318N	Deleterious	Probably damaging	Likely benign	Likely benign	Tolerated	Deleterious	Medium
187	D318G	Deleterious	Benign	Likely benign	Likely disease causing	Tolerated	Deleterious	Medium
188	D318E	Deleterious	Benign	Likely benign	Likely benign	Tolerated	Deleterious	Low
189	I319S	Deleterious	Probably damaging	Likely benign	Likely disease causing	Tolerated	Deleterious	Medium
190	R328W	Deleterious	Benign	Likely benign	Likely benign	Tolerated	Neutral	Low
191	R328Q	Deleterious	Benign	Likely benign	Likely benign	Tolerated	Neutral	Low
192	P330L	Deleterious	Probably damaging	Likely benign	Likely disease causing	Damaging	Deleterious	High
193	R333W	Deleterious	Probably damaging	Likely benign	Likely disease causing	Damaging	Deleterious	High
194	R333Q	Deleterious	Possibly damaging	Likely benign	Likely disease causing	Damaging	Deleterious	High
195	S334R	Deleterious	Possibly damaging	Likely benign	Likely benign	Tolerated	Neutral	Low
196	E335K	Tolerated	Benign	Likely benign	Likely benign	Tolerated	Neutral	Neutral

Table S2. Missense Polβ mutations found in cancer samples.

	AA mutation	Cosmic	cBioportal	Hivebiochemistry
1	M1V			Liver cancer
2	S2I			Blastoma
3	S2R			Liver cancer
4	R4L	Choriocarcinoma		
5	A6S	Small cell lung carcinoma		
6	Q8R	Non small cell lung carcinoma		
7	E9D		Uterine endometrioid carcinoma	Uterine cancer
8	L11F	Glioblastoma		
9	L11V	Colon adenocarcinoma		Uterine cancer
10	G13V		Astrocytoma	Melanoma
11	D17N	Thyroid carcinoma	Papillary thyroid cancer	Malignant glioma
12	M18I	Colon adenocarcinoma, rectum adenocarcinoma	Rectal adenocarcinoma	Colorectal cancer, thyroid carcinoma
13	L22F	Bile duct adenocarcinoma		
14	A23T			Melanoma
15	A23V	Squamous cell lung carcinoma	Lung squamous cell carcinoma	Kidney cancer
16	K27R	Lung adenocarcinoma	Lung adenocarcinoma	Lung cancer
17	N37H	Liver carcinoma		Blastoma, lung cancer
18	A38S			Liver cancer
19	S44C	Stomach adenocarcinoma	Tubular stomach adenocarcinoma	Liver cancer
20	S44F			Stomach cancer
21	Y49S	Prostate adenocarcinoma		Colorectal cancer
22	P50L	Prostate carcinoma		Prostate cancer
23	A59S			Prostate cancer
24	L62F	Thyroid carcinoma		Liver cancer
25	E71K	Endometrioid carcinoma	Uterine serous carcinoma/uteri...	Thyroid carcinoma
26	F76C	Endometrioid carcinoma	Uterine endometrioid carcinoma	Uterine cancer

27	G80R	Serous carcinoma		Uterine cancer
28	R83C	Endometrioid carcinoma, lung adenocarcinoma, stomach adenocarcinoma	Uterine endometrioid carcinoma, lung adenocarcinoma, tubular stomach adenocarcinoma	Germ cell cancer, lung cancer, stomach cancer
29	R83H	Caecum adenocarcinoma		
30	L85V	Esophageal squamous cell carcinoma		
31	E86G	Colon adenocarcinoma		Uterine cancer
32	I88L	Prostate adenocarcinoma		
33	R89W	Endometrioid carcinoma, stomach carcinoma, prostate adenocarcinoma	Uterine endometrioid carcinoma	Melanoma
34	D91E	Malignant melanoma	Cutaneous melanoma	Uterine cancer
35	T93M	Upper aerodigestive tract carcinoma		
36	S95L	Basal cell carcinoma, lung adenocarcinoma		Melanoma
37	R102L	Biliary tract adenocarcinoma		
38	V103I		Esophageal adenocarcinoma	Lung cancer
39	G107C	Stomach adenocarcinoma		Esophageal cancer
40	V115L			Stomach cancer
41	E117Q	Esophageal squamous cell carcinoma		Liver cancer
42	I119M	Prostate adenocarcinoma		Esophageal cancer, prostate cancer
43	L125F	Lung adenocarcinoma	Lung adenocarcinoma	Pancreatic cancer
44	L125I	Endometrioid carcinoma	Uterine endometrioid carcinoma	Lung cancer
45	E129K	Breast carcinoma, cervix squamous cell carcinoma	Breast invasive ductal carcinoma, cervical squamous cell carcinoma	Breast cancer, lung cancer
46	E129D	Lung adenocarcinoma		Uterine cancer
47	D130N	Malignant melanoma		
48	G139A	Breast carcinoma		Cervical cancer
49	R152C	Rectum adenocarcinoma	Rectal adenocarcinoma	Lung cancer
50	R152P	Lung adenocarcinoma		Malignant glioma
51	R152H	Glioma	Oligoastrocytoma	Breast cancer
52	E153G	Glioblastoma		Colorectal cancer

53	D160G	Clear cell renal cell carcinoma		Malignant glioma
54	V162A	Esophageal squamous cell carcinoma		
55	V166L	Ovary serous carcinoma	Serous ovarian cancer	Kidney cancer
56	S171F	Lung squamous cell carcinoma, merkel cell carcinoma		Lung cancer
57	G179S			Esophageal cancer
58	R182G	Malignant melanoma	Lung adenocarcinoma	Lung cancer
59	R182K	Lung adenocarcinoma		Lung cancer
60	G189V			Liver cancer
61	D190A	Prostate adenocarcinoma		
62	D192H	Colon adenocarcinoma	Colon adenocarcinoma	Colorectal cancer, melanoma
63	V193I	Stomach adenocarcinoma	Diffuse type stomach adenocarcinoma	Stomach cancer
64	V193A	Papillary renal cell carcinoma	Papillary renal cell carcinoma	Kidney cancer
65	L194I			Pancreatic cancer
66	V214F	Lung adenocarcinoma	Lung adenocarcinoma	Lung cancer
67	E216K	Prostate adenocarcinoma		
68	E216G	Clear cell renal cell carcinoma		Kidney cancer
69	I224M	Small cell lung carcinoma		Lung cancer
70	M236I			Liver cancer
71	M236V	Large intestine adenocarcinoma		
72	L241F	Endometrioid carcinoma	Uterine endometrioid carcinoma	Uterine cancer
73	N245Y			Stomach cancer
74	P251S	Malignant melanoma, skin squamous cell carcinoma		Melanoma
75	R254I	Non small cell lung carcinoma	Uterine endometrioid carcinoma	Uterine cancer
76	I257V			Liver cancer
77	L259S	Non small cell lung carcinoma	Lung squamous cell carcinoma	
78	L259F	Lung squamous cell carcinoma		Lung cancer
79	Y265H	Breast carcinoma, malignant melanoma	Breast invasive ductal carcinoma	Breast cancer

80	C267F	Esophageal squamous cell carcinoma		
81	G268C	Clear cell renal cell carcinoma, malignant melanoma	Cutaneous melanoma	Kidney cancer
82	L270P	Hepatocellular carcinoma	Hepatocellular carcinoma	
83	G274V	Malignant melanoma	Cutaneous melanoma	
84	I277V		Uterine endometrioid carcinoma	Uterine cancer
85	K280N	Lung squamous cell carcinoma, transitional cell carcinoma	Lung squamous cell carcinoma	Lung cancer
86	N281S	Hepatocellular carcinoma		Blastoma
87	M282I	Cervix squamous cell carcinoma	Cervical squamous cell carcinoma	Cervical cancer
88	R283S	Breast carcinoma		
89	A284V		Uterine endometrioid carcinoma	Uterine cancer
90	A286S			Liver cancer
91	A286V	Large intestine adenocarcinoma		
92	E288K	Breast carcinoma	Breast invasive ductal carcinoma	Breast cancer
93	G290D			Uterine cancer
94	F291L	Upper aerodigestive tract squamous cell carcinoma		
95	Y296D	Small lymphocytic lymphoma		
96	R299C	Large intestine adenocarcinoma		
97	R299S	Upper aerodigestive tract squamous cell carcinoma	Head and neck squamous cell carcinoma	
98	P300L	Malignant melanoma		
99	G305E	Metaplastic breast carcinoma		Breast cancer
100	A307T	Malignant melanoma		
101	E309K	Transitional cell carcinoma		
102	P310L	Malignant melanoma		
103	D314Y	Acute myeloid leukaemia		Hematologic cancer
104	K317I	Malignant melanoma		
105	I319V	Glioma	Astrocytoma	Malignant glioma

106	D321N	Large intestine adenocarcinoma		
107	Y322C	Colon adenocarcinoma		Melanoma
108	W325L	Malignant melanoma	Cutaneous melanoma	
109	R328Q	Malignant melanoma		
110	D332N	Merkel cell carcinoma		
111	R333W	Prostate adenocarcinoma		Prostate cancer
112	R333Q	Caecum adenocarcinoma	Colon adenocarcinoma	Colorectal cancer
